# Dietary Benefits of Pistachio Consumption in Mexico Modeled Using National Health Survey System (ENSANUT) 2012 and 2016 Data

**DOI:** 10.3390/nu17233767

**Published:** 2025-11-30

**Authors:** Alfonso Mendoza Velázquez, Sonia Rodríguez-Ramírez, Ana Elena Pérez Gómez, María Concepción Medina-Zacarias, Leonardo Mendoza Martínez, Adam Drewnowski

**Affiliations:** 1 Center for Research and Economic Studies (CIIE), Department of Economics, Universidad Popular Autónoma del Estado de Puebla (UPAEP), Puebla 72410, Mexico; alfonso.mendoza@upaep.mx (A.M.V.); anaelena.perez@upaep.mx (A.E.P.G.); 2 Center for Nutrition and Health Research (CINyS), National Institute of Public Health, Mexico (Instituto Nacional de Salud Pública), Cuernavaca 62100, Mexico; scrodrig@insp.mx; 3 Center for Evaluation and Surveys Research (CIEE), National Institute of Public Health, Mexico (Instituto Nacional de Salud Pública), Cuernavaca 62100, Mexico; conymedina82@gmail.com; 4 Department of Economics, Universidad de las Américas Puebla, San Andrés Cholula, Puebla 72810, Mexico; lmendozam05@gmail.com; 5 Center for Public Health Nutrition, University of Washington, Seattle, WA 98195, USA

**Keywords:** pistachios, energy-dense snacks, substitution modeling, addition modeling, Mexico Encuesta Nacional de Salud y Nutrición (ENSANUT), nutrient-rich foods (NRF) index, diet quality

## Abstract

Background: Energy-dense non-essential snacks are subject to 8% excise tax in Mexico. Objectives: To model the impact on diet quality of (1) replacing energy-dense snacks with pistachios and (2) adding small amounts of pistachios to the diet. Methods: Data came from the Mexico National Health and Nutrition survey (ENSANUT, by its Spanish acronym) 2012 (*n* = 7132) and 2016 (*n* = 14,764). Dietary intakes were collected using a semi-quantitative food frequency questionnaire. Substitution analyses replaced energy-dense snack foods with equicaloric amounts of pistachios (Model 1) or with mixed nuts/seeds (Model 2). Additional analyses (Model 3) added small amounts of pistachios (10–28 g) to the daily diet. Added sugars, sodium, and saturated fat along with protein fiber, vitamins, and minerals were the main nutrients of interest. Dietary nutrient density was assessed using the Nutrient-Rich Food (NRF9.3) Index. Separate modeling analyses were performed for ENSANUT 2012 and 2016 and for children and adults. Results: Energy-dense foods, mostly sweet, accounted for about 20% of daily energy. Modeled diets with pistachios and mixed nuts/seeds were much lower in added sugars (<8% of dietary energy) and in sodium (<550 mg/day) and were higher in protein, fiber, mono- and polyunsaturated fats, potassium, and magnesium (*p* < 0.05). Significant improvements in dietary quality held across all socio-demographic strata. Adding small amounts of pistachios (10–28 g) to the diet (Model 3) increased calories but also led to better diets and higher NRF9.3 dietary nutrient density scores. Conclusions: Modeled diets with pistachios replacing energy-dense snack foods had less added sugars and sodium and more protein, fiber, vitamins, and minerals. Adding small amounts of pistachios also led to better diets. Pistachios are a healthy snack and can be an integral component of healthy diets.

## 1. Introduction

Tree nuts are nutrient-dense foods with many recognized health benefits [1,2]. Pistachios, almonds, walnuts, and pecans are valuable dietary sources of healthy fats, plant-based proteins, dietary fiber, and a wide range of vitamins and minerals, notably vitamin E, magnesium, zinc, copper, and selenium [3,4,5]. Tree nuts also contain phytosterols and polyphenols with antioxidant properties and well-established benefits for cardiometabolic health [6,7,8,9]. Dietary guidelines in the US and in Mexico recommend consuming more nuts as a part of a healthy, balanced, and sustainable diet [10,11]. Nuts are also prominently featured in the EAT-Lancet Planetary Health Diet [12], with a recommended consumption as high as 50 g per day.

Most nuts, including peanuts, have a high energy density in excess of 275 kcal/100 g [13]. In January 2014, Mexico introduced an 8% excise tax on processed “non-essential energy-dense foods”, defined as those with ≥275 kcal per 100 g [13,14]. The list included chips, cakes, pastries, frozen desserts, sweets, chocolates, and similar snack items. Energy-dense pistachios and other nuts were subject to the 8% tax, if packaged and sold as non-essential, energy-dense foods. The tax covered those tree nuts, ground nuts, and seeds that contained high levels of added sugars, salt, or fat, as well as other ingredients or additives not typically found in whole nuts [13]. The concern was that salted or flavored nuts may have contributed added sugars or salt to the diet [15]. In an effort to reduce the consumption of added sugars, an excise tax was also imposed on beverages with low energy density but high added sugars content [15].

The excise tax was based on a single energy density threshold of ≥275 kcal/100 g and not on nutrient content. Energy density is calculated as calories per 100 g (kcal/100 g), whereas nutrient density is typically calculated in terms of nutrients per 100 g, per 100 kcal, or per serving [13]. In general, energy density and nutrient density are inversely linked. However, tree nuts are an exception to the general rule, being both energy-dense and nutrient-rich [16]. Low-sugar fortified ready-to-eat (RTE) cereals are another exception, since they contain both energy and essential nutrients. The well-established Nutrient-Rich Food (NRF) Index has consistently given high nutrient density scores to tree nuts, including pistachios, almonds, and walnuts [16].

This diet modeling study explored the impact on diet quality metrics of replacing energy-dense foods, many categorized as taxed snacks, with pistachios and with mixed nuts/seeds. The first goal was to identify typical energy-dense snacks that were subject to tax, such as chips, cakes, pastries, frozen desserts, sweets, chocolates, and other such items in the ENSANUT nutrient composition database. To identify populations at greatest need for potential interventions, analyses were conducted across diverse socio-demographic strata. This was carried out to identify subgroups that would most benefit from adopting healthier snacks. Further analyses modeled the likely impact on diet quality of adding small amounts of pistachios to everyday diets of children and adults.

## 2. Materials and Methods

### 2.1. ENSANUT 2012 and 2016 Population Samples

The present analyses were based on two cycles of the Mexican National Health and Nutrition Survey (ENSANUT, by its acronym in Spanish) 2012 and 2016 [17,18]. ENSANUT is the only nationally representative, multistage probabilistic survey of nutrition and health in Mexico. The ENSANUT database contains socio-demographic information and some health data. Analyses were conducted separately for ENSANUT 2012 and 2016 survey waves and separately for children and adults.

Sex was registered as female/male. Socioeconomic status (SES), used by the ENSANUT as an index of household wellbeing, was constructed by analyzing key components related to household characteristics and ownership of household items. For the present analyses, the standardized variable was categorized into SES tertiles (low, medium, and high) for each survey year (2012 and 2016) [19]. The geographic zones were North, Center, South, and Mexico City. Area of residence (urban/rural) was defined according to the number of inhabitants: <2500 for rural and ≥2500 for urban localities. The distribution of respondents who completed a food frequency questionnaire (FFQ) by socio-demographic strata are presented in Table 1 below.

### 2.2. The ENSANUT Food Frequency Questionnaire

Dietary intakes were collected using a previously used and validated semi-quantitative food frequency questionnaire (FFQ) [19]. Participants were excluded if their food consumption exceeded 3 standard deviations (SD) from the mean; if their total energy intake was <500 kcal/day or >5000 kcal/day; or if their protein or fiber intake-to-requirement ratios were greater than 3 SD from the mean, or if their energy intakes were greater than 4 SD from the mean. Pregnant and lactating women were also excluded.

The FFQ instruments used in ENSANUT 2012 and 2016 surveys listed 127 principal food items from multiple food groups. Nutritional data tables used to calculate energy and nutrient intakes are available from the Instituto Nacional de Salud Pública (INSP) at https://insp.mx/informacion-relevante/bam-bienvenida (last access: 29 October 2025).

Relevant to the present modeling study were chips, cakes, pastries, frozen desserts, sweets, chocolates, and other items, many of which had energy density >275 kcal/100 g and fell into the category of taxable non-essential snacks. A full list of taxable FFQ items (provided in Table 2) was established using prior National Health Institute (INSP) papers and government data. Pistachios and other nuts were not a part of the ENSANUT FFQ and were not listed in the FFQ nutrient composition database. Their nutrient composition is provided for comparison purposes.

### 2.3. Nutrient Composition of Pistachio Snacks and Mixed Nuts/Seed Snacks

Composite nutrient profiles for pistachios and for mixed nuts/seeds were created for use in substitution analyses. Those profiles were based on the relative amounts consumed in the 24 h food recalls in ENSANUT 2016. Pistachios were dry roasted pistachios and unsalted pistachios. Mixed nuts were peeled roasted peanuts, pumpkin seeds, pecans, sesame seeds, and almonds. Mean consumption in g/day (shown in Table 3) was as follows: pumpkin seed (Semilla de calabaza): 32.46 g/day; pecan nut (Nuez): 27.74 g/day; sesame seed (Ajonjolí): 5.23 g/day; and almonds (Almendras): 1.57 g/day.

### 2.4. Substitution Modeling (Models 1 and 2) and Addition Modeling (Model 3)

Modeling analyses were conducted to determine the impact of replacing energy-dense snack type foods with isocaloric amounts of pistachios and mixed nuts/seeds on the nutrient content of modeled diets.

Two substitution models were constructed. Substitution Model 1 replaced energy-dense solid snacks with equicaloric amounts of pistachios. Substitution Model 2 replaced energy-dense solid snacks with an equicaloric mix of nuts and seeds as recommended by the Mexico Dietary Guidelines [11]. Beverages and healthy snacks (e.g., nuts, vegetables, fruits) were not replaced. We expected to see improvements in dietary nutrients of public health concern, that is, for reductions in added sugars, sodium, and saturated fat; for higher dietary protein and fiber; and for a higher diet quality overall. We also expected to see greater amounts of polyunsaturated fatty acids, more plant proteins, and higher levels of several vitamins and minerals.

In addition, Model 3 added small amounts (from 10 g to 28 g) of pistachios to the observed diets of teenagers (ages 16–19 y) and adults (age ≥ 20 y). Addition modeling for children respected the Mexican Dietary Guidelines. For the <5 y age group, Model 3 added 10 g of pistachios for both girls and boys. For the 5–11 y age group, Model 3 added 13 g of pistachios to the observed diets for girls and 15 g for boys. For the 12–15 y age group, Model 3 added 18 g of pistachios to diets of girls and 26 g to diets of boys, as recommended by the 2023 Mexican Dietary Guidelines.

### 2.5. Diet-Level Nutrient-Rich Food (NRF) Diet Quality Score

Diet quality was assessed in two ways. First, the observed and modeled diets were compared on the content of added sugars, sodium, and saturated fat. Additional analyses were conducted for the two diets’ content of protein, fiber, and selected vitamins and minerals. Second, the classic diet level NRF9.3 score, calculated for 2000 kcal, was used to assess nutrient density of the observed and the modeled diets. The diet level NRF9.3 score was based on two sub-scores NR9 and LIM. The NR9 sub-score was based on 9 nutrients to encourage: protein, fiber, calcium, iron, potassium, magnesium, vitamin A, vitamin C, and vitamin D. The final score was given by the sum of % percent daily values (DV) of the 9 nutrients to encourage minus the sum of excess (in %) for the 3 nutrients to limit (added sugars, saturated fatty acids, and sodium). In other words, NRF9.3 = NR9 − LIM.

### 2.6. Plan of Analysis

Separate substitution modeling analyses were conducted for ENSANUT 2012 and 2016 and for children and adults. Comparisons, based on *t*-tests and one way ANOVA tests, were made between added sugars and sodium content of the observed and in the isocaloric modeled diets, Models 1 and 2. Further analyses examined protein, fiber, and nutrient content of the observed and modeled diets for the total population and by age group (children, adults). Separate addition modeling analyses (Model 3) were conducted for ENSANUT 2012 and 2016 and for children and adults. Those analyses also examined protein, fiber, and nutrient content of the observed and modeled diets for the total population and by age group (children, adults).

To assess the differential impact of replacing snacks with pistachios or with nuts/seeds across socio-demographic groups, separate modeling analyses were conducted for populations stratified by sex, age group (children ages 0–4 y, 5–11 y, and 12–19 y and adults 20–30 y, 31–50 y, 51–70 y, and >70 y); socioeconomic status (low, medium, and high); and for rural and urban area of residence. Model performance was tested using mean difference *t*-tests with respect to the observed diets with analyses adjusted for the ENSANUT complex survey design. Data provided were means, standard errors (SE), and 95% confidence intervals (CI). Significance level for differences among groups was set at 0.001. All analyses were survey weighted to account for the ENSANUT survey design and reflect the behaviors of the Mexican population. Analyses were conducted in Stata 18 (College Station, TX, USA) statistical package and R-Studio.

## 3. Results

### 3.1. Observed ENSANUT 2012 Data for Children and Adults

**Figure 1:**
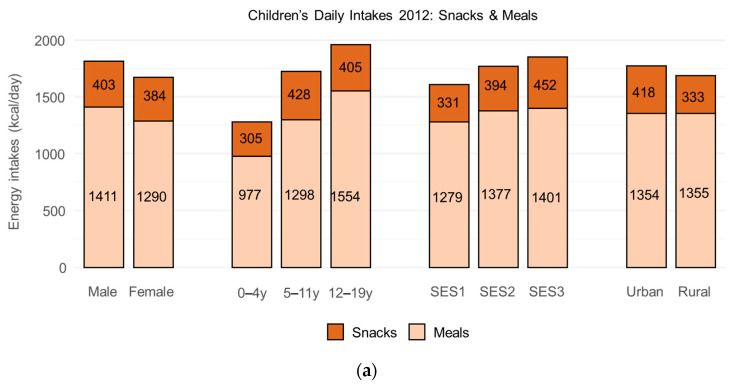

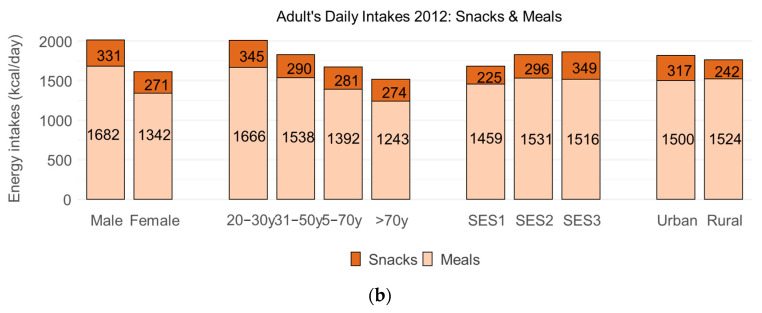
Daily energy intakes by food category for children (**a**) and adults (**b**) by socio-demographic strata in ENSANUT 2012.

ENSANUT 2012 daily energy intakes for children are shown in Figure 1a; the corresponding data for adults are in Figure 1b. Mean energy intake for children was 1750 kcal/day (1815 kcal/day for boys and 1674 kcal/day for girls). Higher daily energy intakes were associated with male sex, higher age group, higher SES, and urban residence (all *p* < 0.001). Mean energy intake for adults was 1806 kcal/day (2013 kcal/day for males and 1614 kcal/day for females). Higher energy intakes were associated with male sex, younger age group, higher SES, and urban residence (all *p* < 0.001).

Energy-dense foods in the taxed snack categories, shown in Figure 1a,b, accounted for 22.7% of dietary energy for children and 16.6% of energy for adults. Calories from energy-dense foods were higher for older children and were associated with higher household incomes and urban residence. Calories from energy-dense foods were higher for younger adults and were associated with higher household incomes and urban residence.

Dietary intakes of energy (kcal/day), added sugars (g/day), and sodium (mg/day) are shown in Table 4. Data are shown separately for children and for adults. Mean intake of added sugars for children was 63.2 g/day (equivalent to 253 kcal energy from added sugars). Mean sodium intake was 2149 mg/day. Higher intakes of added sugars and sodium were associated with male sex, older age group, higher SES, and urban residence (all *p* < 0.001). Mean intake of added sugars for adults was 58.3 g/day (equivalent to 233 kcal energy from added sugars). Mean sodium intake was 2210 mg/day. Higher intakes of added sugars and sodium were likewise associated with male sex, younger age group, higher SES, and urban residence (all *p* < 0.001).

### 3.2. Substitution Modeling (Models 1 and 2) and Addition Modeling (Model 3)

Table 5 shows that the modeled diets in ENSANUT 2012 were lower in added sugars, sodium and saturated fat (all *p* < 0.001) and also lower in carbohydrates (*p* < 0.001). The modeled diets were higher in protein, fiber, potassium, and magnesium (*p* < 0.001). The modeled diets were higher in total fat, mostly MUFA and PUFA, both of which were significantly higher than in the observed diets. The amount of saturated fat was lower in Model 1 and not significantly higher in Model 2. These results for Models 1 and 2 were significant for both children and adults.

Table 6 shows the results for Addition Model 3. Model 3 added pistachios to existing diets of children (1–19 years) and adults (≥20 y). The amounts of pistachios added to the diet depended on age group and were consistent with the Mexican Dietary Guidelines. Model 3 added 28 g of pistachios to the modeled diet for adults; 15 g and 13 g for males and females age 4–19 y; and 10 g of pistachios for children 0–4 y. Table 6 shows that the modeled diets were higher in protein, fiber, MUFA and PUFA, and potassium and magnesium. Since this was addition modeling (as opposed to isocaloric substitution), calories increased, and the amounts of added sugars and sodium remained the same—i.e., addition of pistachios did not increase either added sugars or sodium in the modeled diet. Saturated fat was not significantly higher for children.

**Figure 2:**
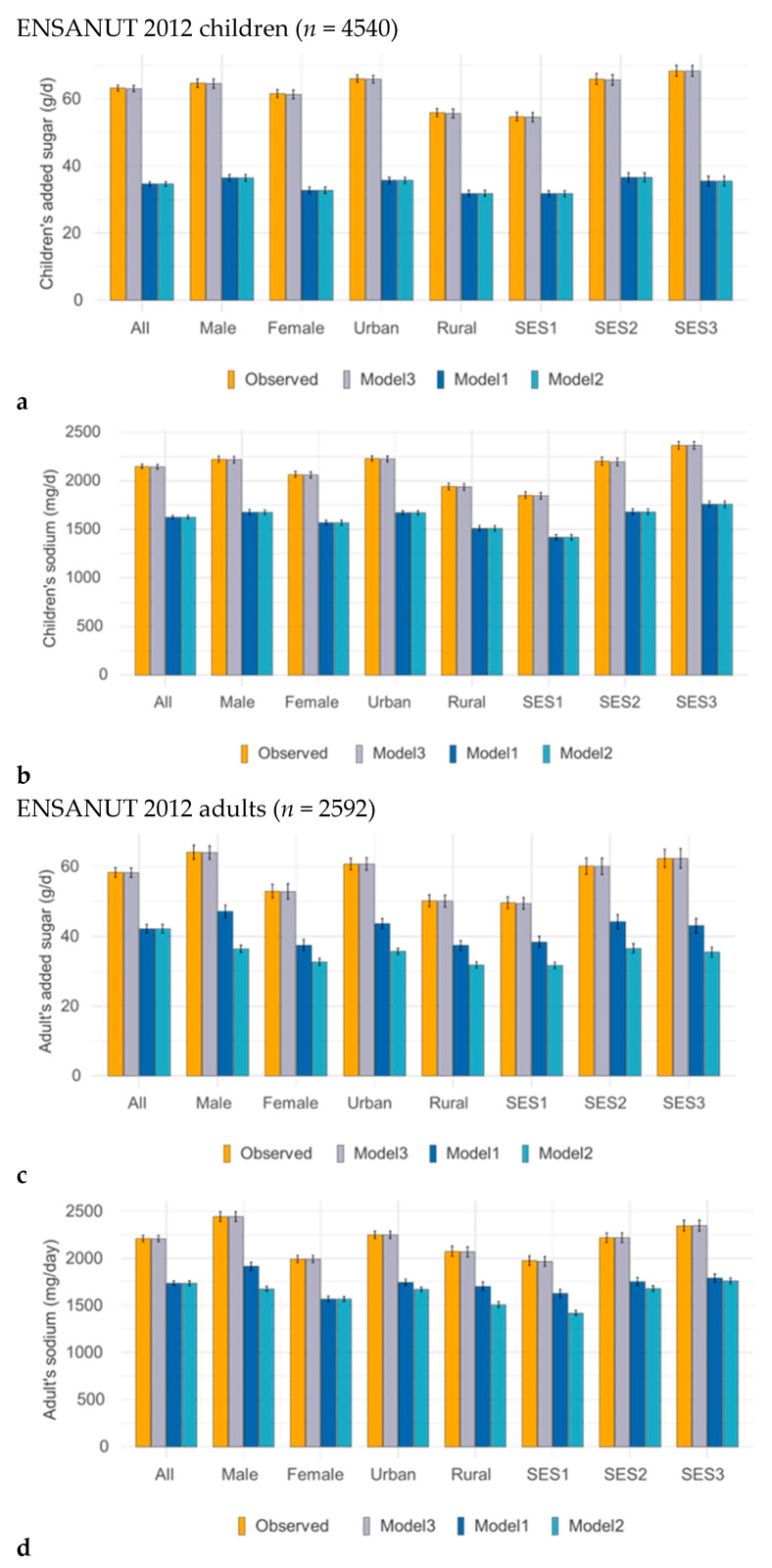
Reductions in added sugars (**a**), sodium (**b**) for children and added sugars (**c**), sodium (**d**) for adults, achieved by isocaloric substitution and addition modeling in ENSANUT 2012 by demographic variables.

Figure 2 shows the reduction in added sugars (a) and sodium (b) for children and adults in Substitution Models 1 and 2 and Addition Model 3 in ENSANUT 2012.

**Figure 3:**
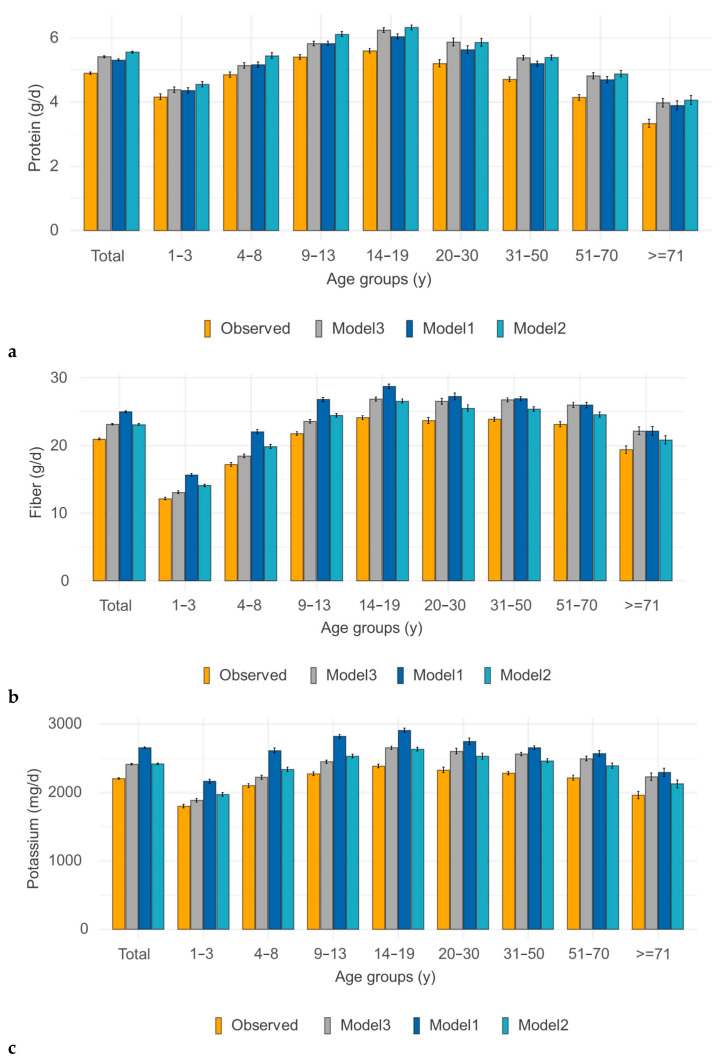

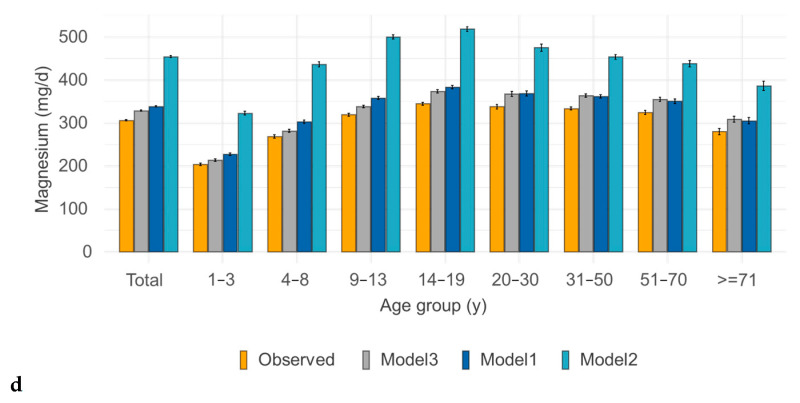
Changes in protein (**a**), fiber (**b**), potassium (**c**), and magnesium (**d**) content of modeled diets from observed consumption by type of model: Model 1 (pistachios), Model 2 (mixed nuts/seeds), and Model 3 (adding 28 g of pistachios). ENSANUT 2012.

Levels of nutrients of interest were changed following isocaloric substitution in modeled ENSANUT 2012 diets. There were observed increases in protein, fiber, and magnesium, as well as in potassium and vitamin E. There was not much change in vitamin C or calcium. There was a slight reduction in vitamin A and iron at younger age—a likely consequence of replacing sweetened cereal-based snacks made with fortified flour. To show how diets improved for all age groups, Figure 3 shows observed and modeled nutrient values for protein, fiber, potassium, and magnesium by age group for both children and adults.

### 3.3. Observed ENSANUT 2016 Data for Children and Adults

**Figure 4:**
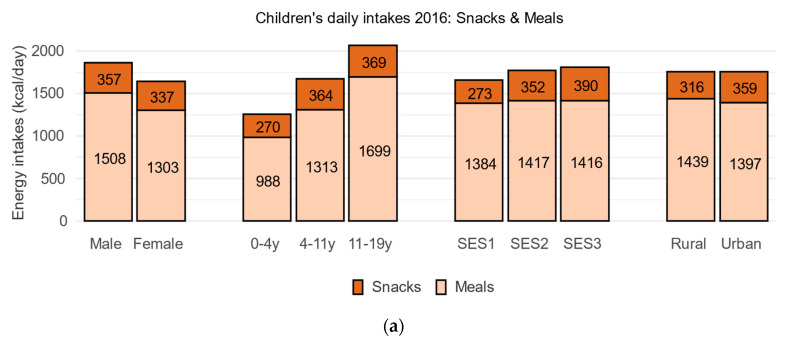

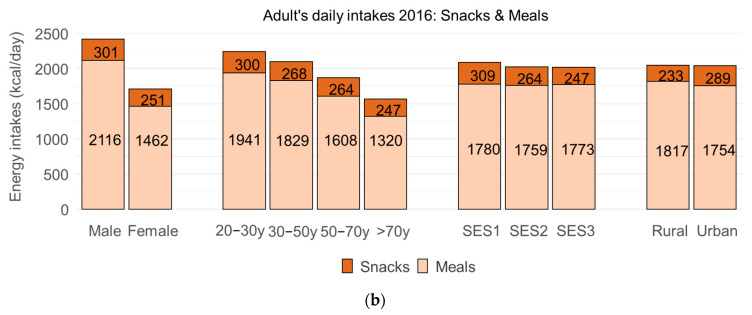
Energy intakes (kcal/day) from meals and solid snacks (see Table 2) in the ENSANUT 2016 data for children (**a**) and adults (**b**).

Figure 4 shows energy intakes by food category for children and adults in ENSANUT 2016. Mean energy intake for children was 1757 kcal (1866 kcal/day for boys and 1641 kcal/day for girls). Higher daily energy intakes were associated with male sex, higher age group, and higher SES (all *p* < 0.001). The urban/rural distinction (prominent in ENSANUT 2012) was no longer observed. Mean energy intake for adults was 2045 kcal/day (2417 kcal/day for males and 1713 kcal/day for females). Higher energy intakes were associated with male sex and younger age groups. Higher SES and urban residence were no longer associated with higher energy intakes. 

Energy-dense foods in the snacks category accounted for 19.8% of dietary energy for children (a decline from 22.7% in 2012) and 14.7% for adults (a decline from 16.6%). Among children, energy-dense foods contributed more energy to diets of older children and were associated with higher household incomes. Among adults, energy-dense foods provided more energy for younger adults and were associated with lower household incomes and rural residence—a reversal of the pattern observed in 2012 ENSANUT.

Table 7 shows dietary intakes of energy (kcal/day), added sugars (g/day), and sodium (mg/day) from ENSANUT 2016. Data are shown separately for children and for adults. Mean intake of added sugars among children was 58.0 g/day (equivalent to 232 kcal energy from added sugars. Mean sodium intake was 2035 mg/day. Higher intakes of sugar among children followed the same trend and the urban/rural distinction was no longer observed. Higher intakes of sodium were associated with male sex, older age group, higher SES, and urban residence (<0.001).

Mean intake of added sugars among adults was 62.7 g/day (251 kcal energy from added sugars). Mean sodium intake was 2270 mg/day. Higher intakes of added sugars among adults were associated with male sex, younger age group, and urban residence but not higher SES. Higher sodium intakes were associated with male sex, younger age, higher SES, and urban residence.

### 3.4. ENSANUT 2016 Substitution Modeling (Models 1 and 2) and Addition Modeling (Model 3)

Table 8 shows that the modeled diets for children in ENSANUT 2016 were not only lower in added sugars and sodium—but also lower in carbohydrates (see Model 1 and Model 2). The modeled diets were higher in protein, fiber, mono- and polyunsaturated fat, potassium, and magnesium. Although total fat was higher, that increase was accounted for by mono- and polyunsaturated fats. The amount of saturated fat was lower in Model 1 and not significantly higher in Model 2.

Table 8 also shows that the modeled diets for adults in ENSANUT 2016 were not only lower in added sugars and sodium—but also lower in carbohydrates (see Model 1 and Model 2). The modeled diets were higher in protein, fiber, mono- and polyunsaturated fat, potassium, and magnesium. Although total fat was higher, that increase was accounted for by mono- and polyunsaturated fats. The amount of saturated fat was lower in Model 1 and not significantly higher in Model 2.

Table 9 shows the results for Addition Model 3, which added pistachios to existing diets of children (1–19 years) and adults (>19 y). The amounts of pistachios followed the Mexican Dietary Guidelines and ranged from 10 g of pistachios for children <4 y to 28 g for adults. Table 9 shows that the modeled diets were higher in protein, fiber, MUFA and PUFA, and potassium and magnesium. Since this was addition modeling (as opposed to isocaloric substitution), calories increased, and the amounts of added sugars and sodium remained the same—i.e., addition of pistachios did not increase either added sugars or sodium in the modeled diet. Saturated fat was not significantly higher for children.

**Figure 5:**
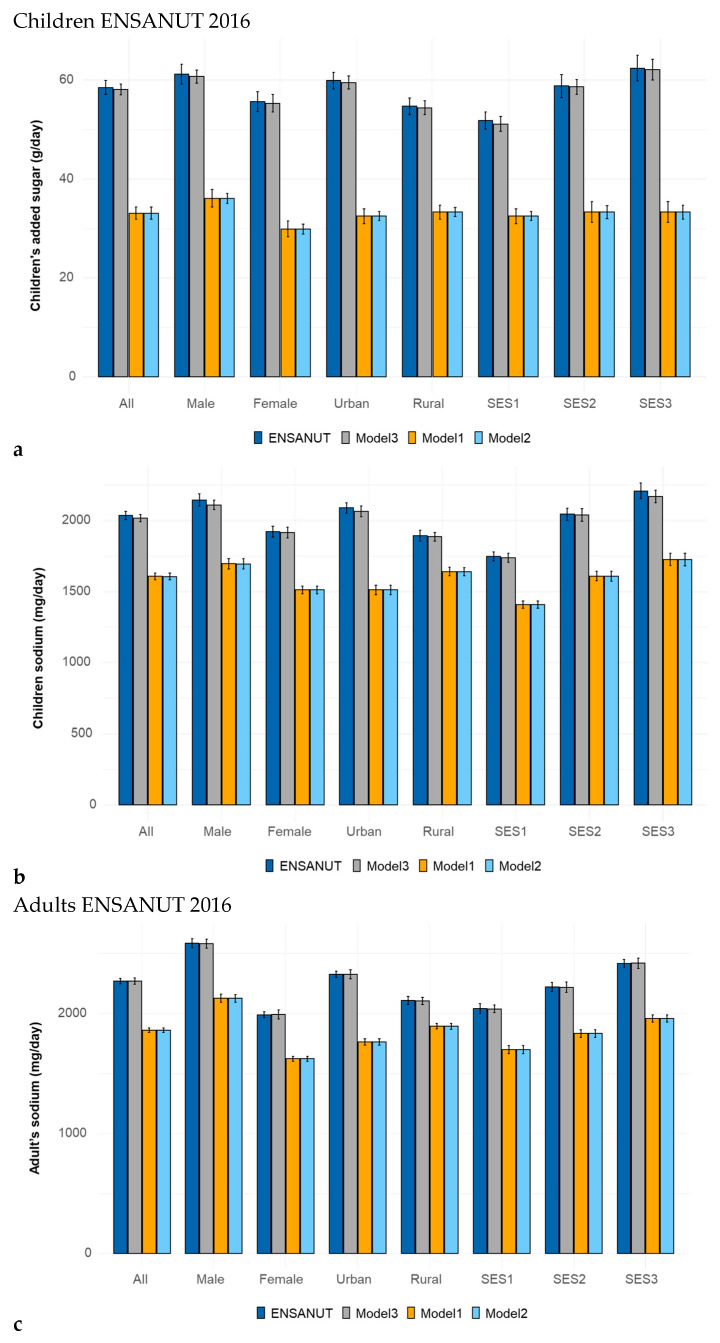

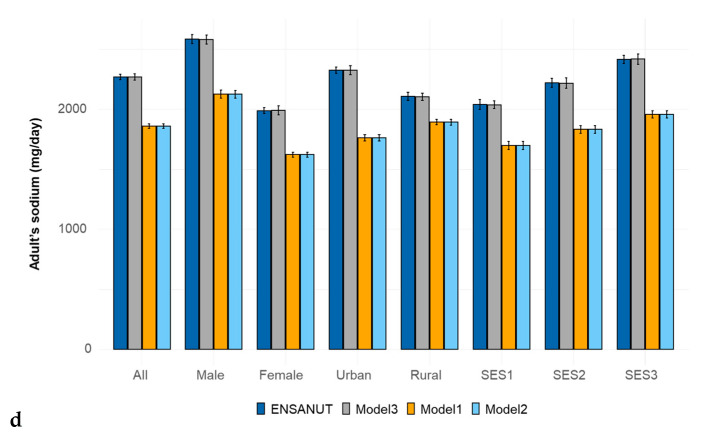
Changes in added sugars (**a**) and sodium (**b**) for children and added sugars (**c**) and sodium (**d**) for adults following isocaloric substitution and addition modeling (Model 1, Model 2, and Model 3) in ENSANUT 2016 by demographic variables. Data are shown separately for children and for adults.

Figure 5 shows the reduction for children in added sugars (a) and sodium (b) and for adults in added sugar (c) and sodium (d) in ENSANUT 2016. Shown are data for observed diets and for Substitution Model 1 and Model 2 and the Addition Model 3.

**Figure 6:**
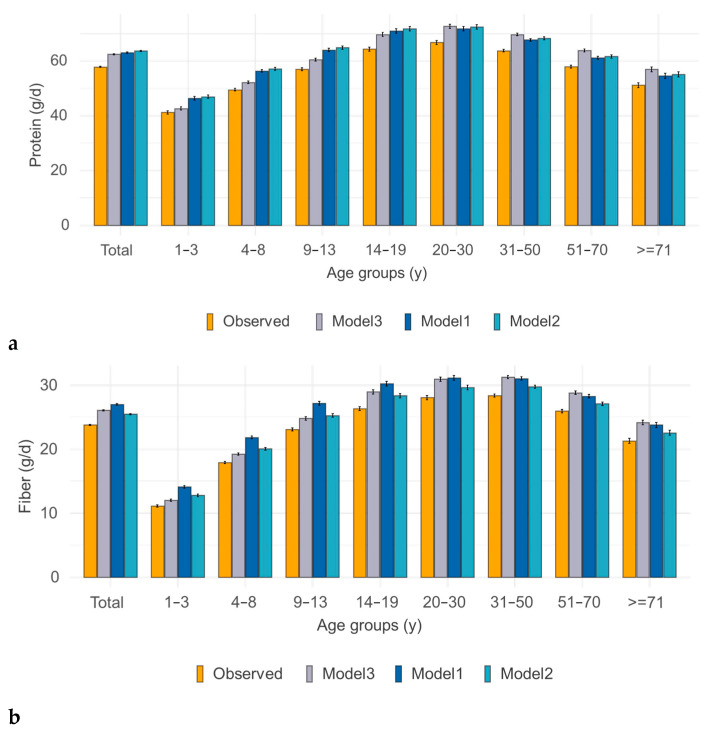

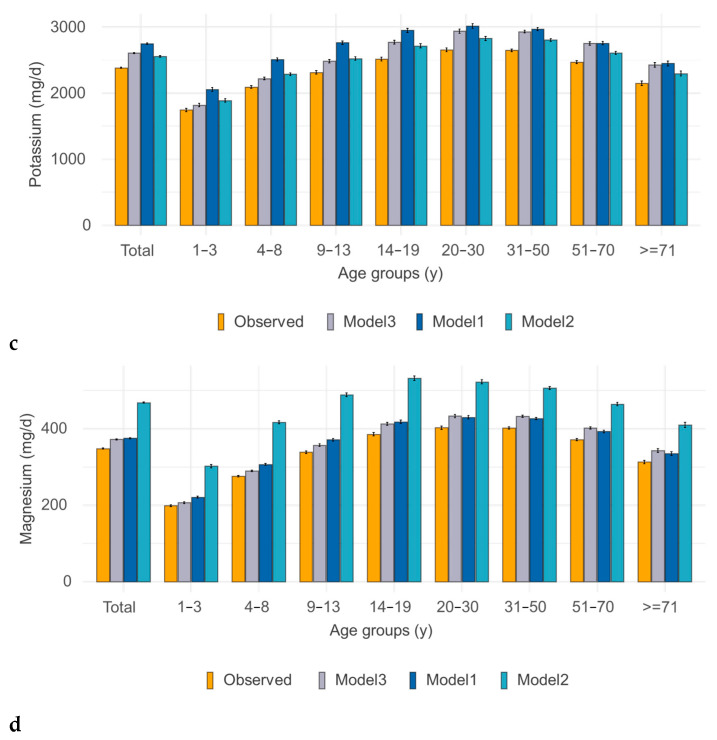
Nutrient intake changes in protein (**a**), fiber (**b**), potassium (**c**), and magnesium (**d**) content of modeled diets from original consumption by type of model: Model 1 (pistachios), Model 2 (mixed nuts), and Model 3 (adding 28 g). ENSANUT 2016.

Figure 6 shows how selected nutrient intakes in modeled ENSANUT 2016 diets changed following isocaloric substitution. There was an increase in protein, fiber, potassium, and magnesium, similar to the results obtained with modeling ENSANUT 2012.

### 3.5. NRF 9.3 Measures of Modeled Dietary Nutrient Density: ENSANUT 2012 and 2016

**Figure 7:**
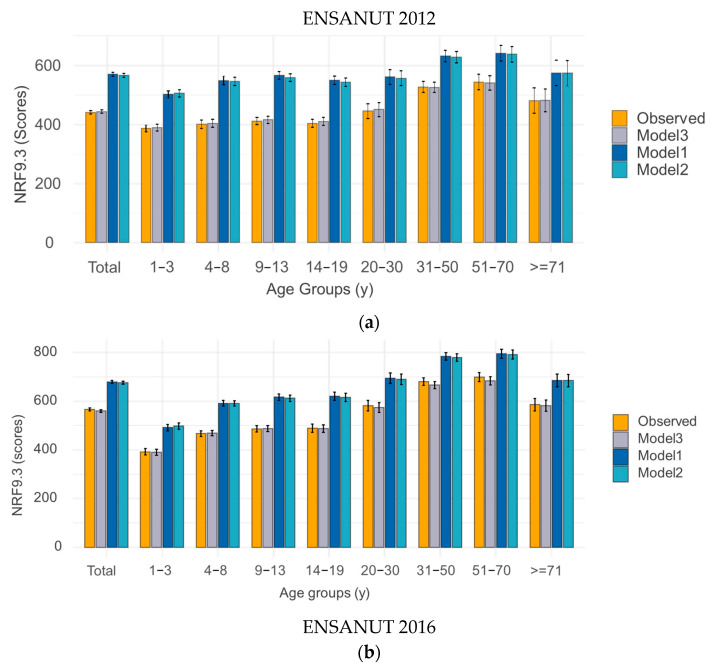
Nutrient-Rich Food Index (NRF9.3) by age group, (**a**) ENSANUT 2012 and (**b**) ENSANUT 2016. Model 1 (pistachios), Model 2 (mixed nuts), and Model 3 (adding 28 g).

The diet level NRF9.3 nutrient density scores for the observed and modeled diets are shown in Figure 7. The data are shown by age group for both children and adults. Separate analyses were conducted for ENSANUT 2012 and 2016 databases.

To measure the total impact of the substitution models on diet quality, the NRF9.3 was examined (see Figure 7). Based on the observed diets for ENSANUT 2012, the population mean value was 441.41 (95% CI 435.03, 447.80). For both models, NRF9.3 mean values were significantly higher, 570.11 for Model 1 (pistachios) and 566.22 for Model 2 (mix). The value for Model 3 (adding 28 g) is 443.94. For all age groups, the NRF9.3 score was significantly higher in both Model 1 and Model 2 as compared to observed diets. The effect was particularly profound among children and adolescents. The values from Model 3 were not significantly different to the observed diets. The results of Model 1 tended to be modestly stronger than for Model 2, but not significantly different.

Similarly, the observed population mean diet value was 566.88 (95% CI 560.63, 573.14). For both models, NRF9.3 mean values were significantly higher, 679.77 for Model 1 (pistachios) and 677.26 for Model 2 (mixed nuts). The value for Model 3 (adding 28 g) is 679.77. As with the ENSANUT 2012 results, the NRF9.3 score for all age groups was significantly higher in both Model 1 and Model 2 as compared to observed diets. Again, the effect was particularly profound among children and adolescents, and the values from Model 3 were not significantly different to the observed diets. The results of Model 1 tended to be modestly stronger than for Model 2, but not significantly different.

The data clearly show significant improvements in diet quality that were achieved by replacing energy-dense foods (very high in added sugars) by pistachios and by mixed nuts and seeds. Greatest improvements were realized in diets of children and adolescents, suggesting that these groups would gain the most from replacing the usual snack foods with pistachios or with mixed nuts/seeds. Similar effects were obtained for ENSANUT 2012 and 2016. Adding small amounts of pistachios to the diet did not lead to modeled diets with more added sugars or sodium. The NRF9.3 scores were not significantly different.

## 4. Discussion

### 4.1. Main Findings

The present analyses of ENSANUT 2012 and 2016 dietary intake data showed that energy-dense solid foods falling in the category of taxed snacks, provided 22.7% of energy in the diets of children and 16.5% in the diets of adults in 2013. By 2016 those percentages were reduced to 19.6% for children and 13.4% for adults. There were also reductions in added sugars from 2012 to 2016: from 14.4% of energy to 13.3% for children and from 12.9% to 12.3% for adults. Sodium intakes remained above 2000 mg/day.

Substitution modeling analyses replaced energy-dense foods with isocaloric amounts of pistachios or mixed nuts/seeds. Modeled diets with pistachios and mixed nuts/seeds were much lower in added sugars (down to <8% of dietary energy) and lower in sodium (<550 mg/day). These improvements were significant for children and adults and held across socio-demographic groups. Diets of children and adolescents were improved the most.

The modeled diets were higher in protein, fiber, mono- and polyunsaturated fats, and potassium and magnesium (*p* < 0.05). These improvements were significant for children and for adults and held across all socio-demographic strata. We did not find a decrease in vitamin C or calcium, since beverages (juice and milk) were not replaced in substitution analyses. On the other hand, Models 1 and 2 (but not Model 3) had less vitamin A. The major sources of vitamin A in Mexico are fortified milk and red and orange vegetables.

Overall diet quality, assessed using the NRF9.3 nutrient density score, was improved across all age groups with the greatest benefits observed for children and for young adults. Similar results were obtained when snacks were replaced with mixed nuts, mostly peanuts and seeds; however, the results for pistachios were stronger.

Adding small amounts of pistachios (from 10 g to 28 g) to the diet (Model 3) also led to higher quality diets as indicated by higher NRF9.3 scores. The additions (no longer isocaloric) were guided by the Mexican dietary guidelines. Even small amounts of pistachios improved diet quality without adding any sugar or sodium.

### 4.2. Pistachios in the Context of the Excise Tax

In January 2014, Mexico implemented an 8% excise tax on non-essential energy-dense foods (NEDFs)—defined as packaged products containing at least 275 kcal per 100 g, such as snacks, cookies, candies, and ice cream [13,14]. The goal was to avoid excess calories, and excess dietary fat, sugar, and salt. The taxed energy-dense snacks also included salted tree nuts, packaged and sold as snacks. Yet unsalted tree nuts including pistachios, are a desirable high-quality snack. They are unique in being both energy-dense and nutrient-rich.

The present analyses provide evidence to support the position that pistachios are a healthy snack that could be used to improve dietary quality overall. Indeed, pistachios could be considered an “essential food” based on Mexico Dietary Guidelines, EAT-Lancet recommendations, and approved health claims. Our modeled diets indicate that pistachios could become a source of macro- and micronutrients that could be incorporated into public food programs. Further investigation is needed to determine the economic accessibility of pistachios, which is traditionally thought to be expensive. Pistachios are good candidates for consumption as minimally processed dried oilseeds with lower probability of generating allergic reactions, compared to peanuts which are widely available and consumed in Mexico [20].

### 4.3. Observed Improvements in the ENSANUT Diets Between 2012 and 2016

The overall quality of diets in Mexico improved between 2012 and 2016. There was a decline in energy intakes from energy-dense foods that was observed among both children and adults. Percent energy from added sugars declined as well. There was less of a socioeconomic gradient in diet quality, suggesting fewer social disparities in access to sufficient dietary energy and to recommended nutrients. Whether this was directly related to the excise tax is hard to determine. Other studies suggest that in the first year after implementation, purchases of taxed foods dropped by about 5.1% per person per month [21]. However, no changes were observed in rural areas, suggesting the tax’s impact is weaker outside urban centers [14]. The present data point to an interesting finding—by 2016 higher consumption of energy-dense snack foods was now associated with lower incomes and rural residence.

### 4.4. What Are the Implications for Public Health Policy?

As of early 2025, Mexico has banned the sale of any products with warning labels in schools, including many taxed items [22]. The warning-label system (black stop-sign seals for high-calorie, sugar, fat, and sodium content) continues to be phased in.

However, the Mexican policy is to promote the consumption of nuts and seeds that are both energy-dense and nutrient-rich. The Mexican general guidelines for the sale of foods in schools, launched in 30 September 2024 [23], which include a table listing the diverse foods allowed in schools: desserts made from seeds and/or whole grains (without added fat or sugar) such as natural popcorn, amaranth dulce de leche (with minimal added sugar), mixed nuts, walnuts, almonds, cranberries, or prunes and snacks made from natural seeds (without salt and without frying) such as peanuts, dehydrated beans and lentils, and pumpkin seeds. The Health Department is preparing to implement the Mexican Dietary Guidelines to further promote the consumption of recommended food groups, including nuts and seeds.

Nuts and seeds are recommended by dietary guidelines in multiple countries [24], including US and Mexico. This has implications for public health guidance and for public health policy. First, nuts and seeds are recommended by all dietary guidelines, including US and Mexico. Tree nuts are high in protein, fiber, and healthy fats and are reported to have positive effects on health [25]. In this study we specifically show that pistachios (unlike typical sweet or salty snacks) have zero added sugars and little sodium. They are also high in protein, fiber, and healthy fats. Tree nuts, including pistachios, belong with healthy snacks and, in terms of Mexican policies, are already viewed as an essential component of a healthy diet.

On the other hand, taxation on food and non-alcoholic beverages are employed to promote healthier diets and combat the high prevalences of obesity and diet-related non-communicable diseases. The evidence shows that in different countries, the taxation of high-fat, salt, or sugar foods had an effect on decreasing their sales, or purchases, and intakes [26]. Mexico is a pioneer in Latin America in implementing these types of policies, finding a positive effect in reducing purchases and consumption of these energy-dense foods. Despite these positive results, another simulation study suggests that these taxes could be higher to further contribute to reducing the prevalence of obesity [27]. If the tax on these foods is higher and larger quantities of them are replaced with nuts and seeds, as well as other healthy foods, it could contribute more quickly to reducing obesity. Helping to include this food group in the daily diet by improving access would have public health benefits.

### 4.5. Limitations

The study had limitations. The semi-quantitative FFQ is a relatively crude tool for assessing habitual diets; however, it is useful for providing snapshots of dietary intakes for large and representative populations. Self-reported dietary intakes suffer from under-reporting and bias. The energy-dense foods, falling into the taxed snacks category, were not necessarily eaten as snacks. Unlike 24 h dietary recalls, FFQ instruments do not capture information about mealtimes and the temporal distribution of intakes. Even though the identified foods were of high energy density and contained added sugars and sodium, specific excise taxes may have varied within product categories. As a result, the present comparison is between energy-dense foods, many falling into the category of non-essential taxed snacks, and pistachios and mixed nuts/seeds. Even though solid energy-dense snacks were replaced with solid energy-dense pistachios, substitution modeling does not take consumer attitudes or individual food preferences into account. Nutrient density analyses were based on the available nutrients; there were no data on other phytochemicals that are present in pistachios and tree nuts. Further investigation is needed on the nutrient quality of pistachios as compared to other competing healthy snacks. Finally, the most recent available ENSANUT data were for 2016; more recent data are expected to become available shortly. Future studies are needed to document the barriers to higher pistachio and walnut consumption, not just the economic ones, and to investigate whether there are differences by population group or some other characteristic. We need to replicate the analyses with more recent dietary data and compare the results with different dietary information collection instruments (frequency of consumption vs. 24-h recall).

## 5. Conclusions

Dietary guidelines in Mexico are moving toward the acceptance of tree nuts as valuable components of a healthy diet. Recommendations for schools already include mixed nuts (walnuts, almond), dried fruits, (cranberries or prunes), peanuts and pumpkin seeds (without salt or fat), and pulses. There is every reason to include pistachios and mixed nuts and seeds on the list of recommended healthy snacks with a potential to improve the quality of the overall diet. A diet based on dietary guidelines, which include nuts and seeds, is necessary to meet the requirements of various vitamins and minerals of the different age groups. Replacing the energy from ultra-processed foods with that from nuts and seeds is a dual-purpose alternative, as it would reduce the consumption of critical nutrients such as sugar, sodium, and added fats and improve the vitamin and mineral profile.

## Figures and Tables

**Table 1:** Participant sample characteristics for ENSANUT 2012 and ENSANUT 2016 and for total 2012–2016 waves by socio-demographic variables.

		2012		2016		Total 2012–2016	
		All	%	All	%	All	%
	All	7132	100.0	14,764	100.0	21,896	100.0
Sex	Male	3402	47.7	6204	42.0	9606	65.1
	Female	3730	52.3	8560	58.0	12,290	83.2
Age	Children	4540	63.7	7038	47.7	11,578	78.4
	Adults	2592	36.3	7726	52.3	10,318	69.9
SES	Tertile 1	2588	36.3	5427	36.8	8015	24.4
	Tertile 2	2369	33.2	4867	33.0	7236	30.6
	Tertile 3	2175	30.5	4470	30.3	6645	45.0
Region	North	1680	23.6	3207	21.7	4887	19.5
	Center	2542	35.6	4949	33.5	7491	34.4
	Mexico City	445	6.2	1556	10.5	2001	15.2
	South	2465	34.6	5052	34.2	7517	30.9
Zone	Urban	4607	64.6	7116	48.2	11,723	74.2
	Rural	2525	35.4	7648	51.8	10,173	25.8

**Table 2:** Nutrient composition of taxed food categories.

ENSANUT Snack Type Foods	Energy Density kcal/100 g	Added Sugars g/100 g	Sodium mg/100 g	Fiber g/100 g	Saturated Fat mg/100 g
1. Specialty cereal	450.0	30.0	350.0	5.0	13.8
2. Chocolate cereal	377.9	36.6	527.0	2.2	1.4
3. Light cereal	352.1	19.8	442.3	6.9	0.7
4. Sweetened flake cereal	372.1	39.4	450.0	1.8	0.2
5. Cereal (Corn Flakes)	377.9	24.8	566.1	3.7	0.2
6. Fruit-flavored cereal	389.8	39.3	528.3	1.7	1.7
7. Assorted cereal varieties	380.4	35.5	518.3	4.5	0.6
8. High-fiber cereal	283.8	18.6	359.3	25.9	0.9
9. Multi-ingredient cereal	388.0	30.3	420.2	4.7	1.6
10. Candied or dried fruits	303.6	28.5	11.4	4.0	0.8
11. White bread	288.6	0.0	1326.0	1.9	0.5
12. Whole wheat bread	266.2	1.1	518.0	5.4	1.0
13. Bakery donuts, churros	452.7	6.9	349.0	0.0	4.8
14. Savory crackers	445.5	6.6	895.1	4.2	5.1
15. Sweet cookies (all types)	434.2	25.7	414.7	2.5	5.7
16. Cereal bars	396.1	24.3	273.7	8.2	6.6
17. Other sweet bread	388.6	13.5	234.6	2.2	9.9
18. Packaged pastries, donuts	410.2	32.3	264.6	1.3	11.8
19. Chocolate	737.1	85.8	85.7	4.8	28.3
20. Flavoring for milk (sugar)	388.7	72.8	187.4	2.7	2.8
21. Spicy candies (tamarind)	166.8	25.2	103.8	1.4	0.1
22. Fried snacks (incl peanuts)	517.4	0.8	797.6	4.4	12.6
23. Popcorn	472.3	0.0	779.3	9.3	8.1
24. Marshmallow lollipops, candies	290.0	48.6	53.4	0.9	2.1
25. Gelatin, flan	73.8	14.1	69.9	0.1	0.6
26. Cake or pie	293.9	35.4	586.4	0.6	8.5
27. Sorbet shaved ice, popsicles	76.6	16.6	26.0	0.0	0.1
28. Ice cream, milk-based popsicles	179.0	19.3	71.0	1.3	5.4
29. Candy (candies, lollipops)	358.5	66.9	272.4	0.2	0.0
Foods not in ENSANUT FFQ					
Pistachios (plain, dry roasted)	562.0	0.0	1.0	10.3	5.6
Almonds (plain, dry roasted)	579.0	0.0	1.0	12.5	4.0
Peanuts (dry roasted, unsalted)	567.0	0.0	6.0	8.5	6.8
Peanuts (salted)	567.0	0.0	400–600	8.5	6.8

**Table 3:** Weights used to develop nutrient profiles for replacement snacks in Model 1 (pistachios) and Model 2 (mixed nuts/seeds).

		Weight (g) *	Energy Density **
Pistachios	Pistachios, plain dry roasted, unsalted	100.0	5.72
Nuts/seeds	Peanuts (Cacahuate tostado sin cascara)	32.98	5.87
	Pumpkin seeds (Semilla de calabaza)	32.46	5.59
	Pecan nuts (Nuez)	27.75	6.54
	Sesame seeds (Ajonjolí)	5.23	5.73
	Almonds (Almendras)	1.57	5.79

* Weights from 24 h recall ENSANUT 2016. Out of the 4000 individuals in this 24 h recall none report any pistachio consumption. In 2012, out of 10,000, only 4 people report pistachio consumption. ** Energy density is obtained from nutrition tables and is calculated as kcal/g.

**Table 4:** Energy (kcal/day), added sugars (g/day), and sodium (mg/day) in observed diets of children and adults by socio-demographics. ENSANUT 2012.

		Kcal/day			Added Sugars g/day			Sodium mg/day		
		Mean	SE	CI	Mean	SE	CI	Mean	SE	CI
Children < 20 y (*n* = 4540)										
	All	1749	14.2	1722, 1777	63.2	0.9	61.4, 64.9	2149	22.9	2104, 2193
Sex	Male	1815	20.3	1775, 1855	64.6	1.3	62.1, 67.2	2222	33.2	2157, 2287
	Female	1675	19.7	1636, 1714	61.5	1.2	59.1, 64	2065	30.9	2005, 2126
	*p* <	0.001			0.102			0.001		
Age	0–4 y	1283	19.1	1245, 1320	19.4	0.7	18, 20.7	1186	21.3	1145, 1228
	5–11 y	1727	23.2	1682, 1773	29.2	0.8	27.7, 30.7	1556	24.4	1508, 1603
	12–19 y	1960	22.9	1915, 2005	45.2	1.4	42.5, 47.9	1861	30.8	1801, 1921
	*p* <	0.001			0.001			0.001		
SES	Tertile 1	1611	23.8	1564, 1657	54.7	1.3	52.1, 57.3	1851	34.6	1783, 1919
	Tertile 2	1771	24	1724, 1818	65.9	1.6	62.7, 69.1	2204	39.8	2126, 2282
	Tertile 3	1854	24.7	1806, 1903	68.3	1.6	65.1, 71.4	2366	40.2	2287, 2445
	*p* <	0.001			0.001			0.001		
Area	Urban	1773	17.5	1739, 1807	66	1.1	63.8, 68.3	2229	28.3	2174, 2285
	Rural	1689	22.9	1644, 1734	55.8	1.2	53.3, 58.2	1941	35.6	1871, 2011
	*p* <	0.007			0.001			0.001		
Adults ≥ 20 y (*n* = 2592)										
	All	1806	20	1767, 1846	58.3	1.4	55.5, 61.0	2210	32.5	2146, 2273
Sex	Male	2013	31.6	1951, 2075	64.1	2	60.2, 67.9	2442	51.6	2341, 2544
	Female	1614	21.8	1571, 1656	52.9	2	49.0, 56.8	1993	37.5	1919, 2066
	*p* <	0.001			0.001			0.001		
Age	20–30 y	2012	46.8	1920, 2103	49	2.8	43.6, 54.4	1963	60.2	1845, 2081
	31–50 y	1828	28.9	1771, 1885	42.2	2	38.4, 46.0	1762	38.6	1686, 1838
	51–70 y	1674	33	1609, 1739	37.5	1.8	33.9, 41.1	1581	48.4	1486, 1676
	>70 y	1518	65.5	1389, 1647	35.6	3.9	28.0, 43.3	1416	88.1	1243, 1589
	*p* <	0.001			0.001			0.001		
SES	Tertile 1	1685	29.7	1626, 1743	49.6	1.7	46.3, 52.9	1975	52	1873, 2077
	Tertile 2	1828	34.1	1761, 1895	60.1	2.3	55.5, 64.7	2220	51.6	2119, 2322
	Tertile 3	1865	34.7	1797, 1933	62.3	2.6	57.2, 67.3	2345	57.6	2232, 2458
	*p* <	0.001			0.001			0.001		
Area	Urban	1818	23.8	1772, 1865	60.7	1.7	57.3, 64.1	2250	39	2173, 2326
	Rural	1767	34.1	1700, 1834	50.2	1.7	46.9, 53.5	2076	54.9	1968, 2183
	*p* <	0.278			0.001			0.014		

SE: standard errors; CI: confidence intervals.

**Table 5:** ENSANUT 2012. The observed and modeled data of children (1–19 years) and adults (≥20 y). Substitution Models 1 and 2.

	Observed		Model 1			Model 2			
	Mean	CI	Mean	CI	*p*<	Mean	CI	*p*<	Comment
Children < 20 y (*n* = 4540)									
Added sugars (g/d)	63.2	61.4, 64.9	34.6	33.2, 36.0	0.001	34.6	33.2, 36.0	0.001	Lower
Sodium (mg/d)	2149	2104, 2193	1625	1591, 1660	0.001	1625	1590, 1659	0.001	Lower
Saturated fat (g/d)	22.7	22.2, 23.2	21.1	20.7, 21.5	0.001	22.2	21.7, 22.6	0.001	Lower
Carbohydrates (g/d)	258.4	254.2, 262.7	209.6	206, 213.2	0.001	200.6	197.1, 204.1	0.001	Lower
Protein (g/d)	55.5	54.6, 56.4	63.7	62.6, 64.8	0.001	64.6	63.5, 65.7	0.001	Higher
Total fat, g/d	57.7	56.5, 58.8	77.8	76.3, 79.4	0.001	81.9	80.2, 83.5	0.001	Higher
Monounsaturated fat (g/d)	18.1	17.7, 18.5	30.2	29.6, 30.9	0.001	25.1	24.6, 25.6	0.001	Higher
Polyunsaturated fat (g/d)	13	13.1, 13.8	20.0	19.6, 20.5	0.001	27	26.4, 27.6	0.001	Higher
Fiber (g/d)	20.3	19.9, 20.7	25.1	24.6, 25.6	0.001	22.9	22.4, 23.3	0.001	Higher
Magnesium (mg/d)	302	297, 307	338	333, 344	0.001	473	465, 481	0.001	Higher
Potassium (mg/d)	2238	2197, 2278	2759	2711, 2808	0.001	2482	2438, 2526	0.001	Higher
Adults ≥ 20 y (*n* = 2592)									
Added sugars (g/d)	58.3	55.5, 61.0	42.1	39.8, 44.5	0.001	42.1	39.8, 44.5	0.001	Lower
Sodium mg/d	2209	2146, 2273	1735	1684, 1786	0.001	1734	1684, 1785	0.001	Lower
Saturated fat (g/d)	21.4	20.8, 22.1	20	19.4, 20.6	0.001	20.8	20.2, 21.4	0.001	Lower
Carbohydrates (g/d)	266.6	260.7, 272.4	230.8	225.7, 235.8	0.001	223.9	219, 228.8	0.001	Lower
Protein (g/d)	59	57.7, 60.3	64.2	62.7, 65.6	0.001	64.9	63.4, 66.4	0.001	Higher
Total fat, g/d	56.4	54.7, 58.1	71.4	69.3, 73.6	0.001	74.5	72.3, 76.7	0.001	Higher
Monounsaturated fat (g/d)	17.9	17.4, 18.5	27.2	26.3, 28.1	0.001	23.3	22.6, 2	0.001	Higher
Polyunsaturated fat (g/d)	13.5	12.9, 14.0	18.5	17.9, 19.1	0.001	23.8	23, 24.6	0.001	Higher
Fiber (g/d)	23.2	22.7, 23.8	26.7	26.1, 27.3	0.001	25	24.5, 25.5	0.001	Higher
Magnesium (mg/d)	330	324, 337	361	354, 369	0.001	464	453, 475	0.001	Higher
Potassium (mg/d)	2310	2259, 2361	2722	2661, 2784	0.001	2511	2456, 2567	0.001	Higher

CI: confidence interval.

**Table 6:** ENSANUT 2012. Addition Model 3 observed and modeled data of children (1–19 y) and adults (>19 y).

	Children (1–19 y)					Adults ≥ 20 y					
	Observed		Model 3 (*n* = 4559) *			Observed		Model 3 (*n* = 2595)			Comment
	Mean	CI	Mean	Cl	*p*<	Mean	Cl	Mean	Cl	*p*<	
Total kcal/day	1750	1720, 1779	1855	1825, 1885	0.001	1806	1763, 1850	1966	1922, 2009	0.001	Higher
Added sugars, g/d	63.2	61.4, 64.9	63	61.2, 64.8	−	58.3	55.5, 61.0	58.2	55.4, 61.0	−	Lower
Sodium, mg/d	2149	2104, 2193	2144	2097, 2190	0.001	2210	2146, 2273	2208	2140, 2276	0.001	Lower
Total fat, g/d	57.7	56.5, 58.9	66	64.7, 67.3	0.001	56.4	54.7, 58.1	69.2	67.4, 70.9	0.001	Higher
Saturated fat, g/d	22.7	22.2, 23.2	23.7	23.1, 24.2	0.001	21.4	20.8, 22.1	23	22.3, 23.7	0.001	Lower
Monounsaturated fat, g/d	18.1	17.7, 18.5	22.6	22.2, 23.0	0.001	17.9	17.4, 18.5	24.8	24.2, 25.3	0.001	Higher
Polyunsaturated fat, g/d	13	13.1, 13.8	15.9	15.5, 16.2	0.001	13.5	12.9, 14.0	17.2	16.7, 17.7	0.001	Higher
Protein, g/d	55.5	54.6, 56.4	59.2	58.2, 60.3	0.001	59	57.7, 60.3	64.8	63.4, 66.2	0.001	Higher
Carbohydrates, g/d	258.4	254.2, 262.7	262.9	258.4, 267.4	0.001	266.6	260.7, 272.4	274.1	267.5, 280.7	0.001	Lower
Fiber, g/d	20.3	19.9, 20.7	22.2	21.7, 22.6	0.001	23.2	22.7, 23.8	26.1	25.6, 26.6	0.001	Higher
Magnesium, mg/d	302	297, 307	321	316, 327	0.001	330	324, 337	361	353, 368	0.001	Higher
Potassium, mg/d	2238	2197, 2278	2419	2377, 2461	0.001	2310	2259, 2361	2589	2533, 2645	0.001	Higher

* Model 3, *n* = 4559 children after exclusions. Model 3, *n* = 2595 adults after exclusions. CI: confidence interval.

**Table 7:** ENSANUT 2016: Characteristics of observed diets for children and adults.

		Kcal/day			Added Sugars			Sodium		
		Mean	SE	CI	Mean	SE	CI	Mean	SE	CI
Children < 20 y (*n* = 7038)										
	All	1757	20.9	1716, 1798	58.5	1.2	56.2, 60.8	2035	33.8	1968, 2101
Sex	Male	1866	24.3	1819, 1914	61.2	1.4	58.5, 63.9	2143	39.3	2065, 2220
	Female	1641	29	1584, 1698	55.6	1.8	52, 59.2	1921	42	1838, 2003
	*p*<	0.001			0.012			0.001		
Age	0–4 y	1258	25.4	1208, 1308	42	1.6	38.7, 45.2	1436	30.1	1377, 1495
	4–11 y	1678	30.4	1618, 1738	55.3	2.1	51.3, 59.4	1956	41.6	1874, 2038
	11–19 y	2069	30.1	2010, 2128	69.3	1.7	65.8, 72.7	2394	54.7	2287, 2502
	*p*<	0.001			0.001			0.001		
SES	Tertile 1	1643	31.7	1581, 1706	51.8	1.9	48.1, 55.5	1747	40.7	1667, 1827
	Tertile 2	1757	30.7	1697, 1818	58.8	1.7	55.5, 62.1	2042	45.2	1953, 2131
	Tertile 3	1827	35.6	1757, 1897	62.4	2.2	57.9, 66.8	2206	59.7	2088, 2324
	*p*<	0.001			0.001			0.001		
Zone	Rural	1756	32.4	1692, 1820	54.7	1.7	51.3, 58.1	1891	52.6	1788, 1995
	Urban	1757	25.9	1706, 1808	59.9	1.5	57, 62.7	2087	41.8	2005, 2169
	*p*<	0.975			0.023			0.004		
Adults ≥ 20 y (*n* = 7726)										
	All	2045	20.8	2004, 2086	62.7	1.3	60.2, 65.3	2270	26.9	2217, 2323
Sex	Male	2418	33.9	2351, 2484	76.5	2.1	72.3, 80.7	2584	41.1	2504, 2665
	Female	1713	18.1	1678, 1749	50.4	1.4	47.6, 53.2	1989	24.3	1941, 2037
	*p*<	0.001			0.001			0.001		
Age	20–30 y	2263	39.8	2184, 2341	77	3	71.1, 82.9	2465	50.3	2366, 2564
	30–50 y	2094	30.8	2033, 2154	62.8	1.7	59.5, 66.2	2350	35.6	2280, 2420
	50–70 y	1889	34.5	1821, 1957	53.7	2.3	49.2, 58.1	2084	44.8	1996, 2172
	>70 y	1568	39.4	1490, 1645	44.3	2.5	39.4, 49.2	1795	48.9	1699, 1892
	*p*<	0.001			0.001			0.001		
SES	Tertile 1	2006	42.4	1922, 2089	58.9	2.4	54.2, 63.6	2040	49.7	1942, 2138
	Tertile 2	2010	30.4	1950, 2070	62.8	1.7	59.4, 66.3	2219	40.6	2139, 2299
	Tertile 3	2088	29.2	2030, 2145	64.6	2	60.6, 68.5	2417	35.7	2346, 2487
	*p*	0.197			0.155			0.001		
Zone	Rural	2050	44.7	1962, 2138	56.3	2.5	51.4, 61.1	2107	46.7	2015, 2199
	Urban	2044	23.4	1998, 2090	64.9	1.5	61.9, 67.9	2325	32	2262, 2388
	*p*<	0.784			0.008			0.001		

SE: standard error of the mean. CI: confidence interval.

**Table 8:** ENSANUT 2016. The observed and modeled data of children (1–19 years) and adults (≥20 y).

	Observed		Model 1			Model 2			
	Mean	CI	Mean	CI	*p*	Mean	CI	*p*	Comment
Children < 20 y									
Total fat, g/d	59.2	57.3, 61.1	76.3	73.9, 78.8	0.001	79.9	77.3, 82.5	0.001	higher
Saturated fat g/d	23.2	22.4, 24	21.4	20.7, 22.1	0.001	22.4	21.6, 23.1	0.001	lower
Monounsaturated fat g/d	18.2	17.6, 18.8	28.7	27.7, 29.8	0.001	24.2	23.4, 25.1	0.001	higher
Polyunsaturated fat g/d	13.9	13.4, 14.4	19.7	19, 20.3	0.001	25.8	24.9, 26.8	0.001	higher
Protein g/d	57.7	56, 59.3	64.9	63, 66.8	0.001	65.8	63.8, 67.7	0.001	higher
Carbohydrates g/d	254.5	248.9, 260.1	212.8	208.1, 217.6	0.001	204.9	200.3, 209.5	0.001	lower
Added sugars g/d	58.5	56.2, 60.8	33.1	31.6, 34.6	0.001	33.1	31.6, 34.6	0.001	lower
Fiber g/d	20.3	19.7, 20.8	24.4	23.8, 25.1	0.001	22.5	21.9, 23.1	0.001	higher
Magnesium mg/d	315.4	308, 323	348	340, 357	0.001	467	455, 480	0.001	higher
Potassium mg/d	2291	2231, 2352	2745	2670, 2821	0.001	2501	2434, 2568	0.001	higher
Sodium mg/d	2035	1968, 2101	1605	1554, 1656	0.001	1604	1553, 1655	0.001	lower
Adults ≥ 20 y									
Total fat, g/d	62.8	61.2, 64.3	76.3	74.3, 78.2	0.001	79.1	77, 81.1	0.001	higher
Saturated fat g/d	22.7	22.1, 23.4	21.1	20.5, 21.7	0.001	21.8	21.3, 22.4	0.001	lower
Monounsaturated fat g/d	20.2	19.7, 20.8	28.6	27.8, 29.5	0.001	25.1	24.4, 25.8	0.001	higher
Polyunsaturated fat g/d	15.7	15.2, 16.1	20.4	19.9, 21	0.001	25.3	24.6, 26	0.001	higher
Protein g/d	67.2	66, 68.5	71.9	70.5, 73.3	0.001	72.6	71.2, 73.9	0.001	higher
Carbohydrates g/d	299.7	293.4, 306	267.5	261.8, 273.1	0.001	261.2	255.6, 266.7	0.001	lower
Added sugars g/d	62.7	60.2, 65.3	47.4	45.2, 49.6	0.001	47.4	45.2, 49.6	0.001	lower
Fiber g/d	27.4	26.8, 28.1	30.5	29.7, 31.2	0.001	29	28.2, 29.7	0.001	higher
Magnesium mg/d	400	392, 409	428	419, 437	0.001	5228	510, 534	0.001	higher
Potassium mg/d	2732	2674, 2789	3102	3035, 3169	0.001	2909	2847, 2971	0.001	higher
Sodium mg/d	2270	2217, 2323	1859	1814, 1904	0.001	1859	1814, 1904	0.001	lower

CI: confidence interval.

**Table 9:** ENSANUT 2016. The observed and Model 3 (addition) diets of children (1–19 years) and adults (>19 y).

	Children < 20 y (*n* = 7085)					Adults ≥ 20 y (*n* = 7717)					
	Observed		Model 3			Observed		Model 3			Comment
	Mean	CI	Mean	CI	*p*	Mean	CI	Mean	CI	*p*	
Total kcal/day	1750.5	1712, 1789.1	1853.5	1814.5, 1892.6	0.001	2041.3	2000.5, 2082.1	2201.4	2160.7, 2242.2	0.001	higher
Total fat, g/d	59	57.3, 60.7	67.2	65.5, 69.0	0.001	62.7	61.2, 64.2	75.5	74.0, 77.1	0.001	higher
Saturated fat (g/d)	23.1	22.4, 23.9	24.2	23.4, 24.9	0.001	22.7	22.1, 23.4	24.3	23.6, 24.9	0.001	lower
Monounsaturated fat (g/d)	18.1	17.6, 18.7	22.5	22.0, 23.1	0.001	20.2	19.7, 20.8	27.1	26.5, 27.7	0.001	higher
Polyunsaturated fat (g/d)	13.8	13.4, 14.3	16.2	15.7, 16.7	0.001	15.6	15.2, 16.0	19.4	19.0, 19.8	0.001	higher
Protein (g/d)	57.4	56; 58.9	61.2	59.7, 62.7	0.001 0.001	67.2	65.9, 68.4	73.1	71.8, 74.3	0.001	higher
Carbohydrates (g/d)	253.7	248.1, 259.2	258.8	253.2, 264.4	0.001	299.2	292.9, 305.4	307.1	300.9, 313.4	0.001	lower
Added sugars (g/d)	58.3	56, 60.6	58.3	56, 60.6	–	62.5	60, 65.1	62.5	60, 65.1	–	same
Fiber (g/d)	20.2	19.7, 20.8	22.1	21.5, 22.7	0.001	27.4	26.7, 28.1	30.3	29.6, 31	0.001	higher
Magnesium (mg/d)	314.7	307.1, 322.3	334.3	326.6, 342	0.001	399.7	391.4, 408	430.2	421.9, 438.5	0.001	higher
Potassium (mg/d)	2284.0	2224.8, 2343.2	2465.3	2405.3, 2525.2	0.001	2727.6	2670.6, 2784.7	3009.6	2952.5, 3066.7	0.001	higher
Sodium (mg/d)	2019.70	1965.4, 2073.9	2020.7	1966.5, 2075	0.001	2267.5	2214.6, 2320.4	2269.2	2216.3, 2322.1	0.001	lower

CI: confidence interval.

## Data Availability

Publicly available datasets were analyzed in this study. This data is described in the following: ISSN 0036-3634 and doi:10.21149/8593. The original contributions presented in the study are included in the article, further inquiries can be directed to the corresponding author.
